# VEILND (Video Endoscopic Inguinal Lymph Node Dissection) with Florescence Indocyanine Green (ICG): A Novel Technique to Identify the Sentinel Lymph Node in Men with ≥pT1G2 and cN0 Penile Cancer

**DOI:** 10.1155/2021/5575730

**Published:** 2021-10-29

**Authors:** Milan Hora, Ivan Trávníček, Štěpánka Nykodýmová, Jiří Ferda, Denisa Kacerovská, Květoslava Michalová, Ondřej Hes, Suks Minhas

**Affiliations:** ^1^Department of Urology, University Hospital Plzeň and Faculty of Medicine in Plzeň, Charles University, Pilsen, Czech Republic; ^2^Department of Radiology, University Hospital Plzeň and Faculty of Medicine in Plzeň, Charles University, Pilsen, Czech Republic; ^3^Department of Pathology, University Hospital Plzeň and Faculty of Medicine in Plzeň, Charles University, Pilsen, Czech Republic; ^4^Imperial College Healthcare, London, UK

## Abstract

**Introduction:**

In men with ≥pT1G2 cN0, penile cancer lymph node sampling is recommended with either (1) scintigraphically labelled Dynamic sentinel lymph node biopsy (DSLNB) or (2) modified inguinal lymph node dissection (MILND). Although DSLNB is a minimally invasive technique, the false negative rate can be about 10%, and a further operative procedure is required if positive. Open MILND is a diagnostic and therapeutic option but has a much higher morbidity. A potential compromise is the technique of LND-VEILND (video endoscopic inguinal LND) that can be combined with ICG florescence marking of sentinel lymph node (SLN). We present a pilot study of ICG-VEILND. The aim was to validate the applicability of a combination ICG marking of SLN in VEILND (to increase probability to excise SLN) and determine the optimal timing and dosage of ICG.

**Materials and Methods:**

15 patients with VEILND (24 groins) underwent ICG application with fluorescence near-infrared (NIR 803⟶830 nm) detection. ICG is applied subcutaneously adjacent to the penile cancer or residual stump of penis or suprapubic region (in a history of total penectomy: 5 cases). The dose of 1.25 mg (ICG) was applied in one case with invisible SLN, the dose of 2.5 mg in 1 mL in 8 cases, and 5 mg in the remaining 6 patients (10 groins).

**Results:**

Failure of marking SLN with ICG occurred in 25.0% of cases (6/24): due to application of 1.25 mg ICG, extensive metastasis to SLN, in 4 cases, the cause was unknown (16.7%, 4/24). In the short follow-up period, no local recurrence was seen in the pN0 ICG group.

**Conclusion:**

Fluorescence infrared image with ICG dye increases the probability of removal of the SLN during VEILND. The dose of ICG is 2.5 (5) mg diluted in 1 ml and can be applied preoperatively even in the suprapubic region in men with a history of total penectomy, with an unexplainable failure of ICG marking in 16.7%.

## 1. Introduction

Lymph node metastasis is the single most important prognostic factor determining survival in men with penile cancer. An integral part of the management of penile cancer is the detection of occult metastases to the inguinal lymph nodes (ILNs). Compared to delayed excision, early excision of lymph node (LN) metastases improves cancer-specific survival among patients with penile cancer (84% vs. 35% at 3 yrs) [1]. In men with palpable ILNs, radical inguinal lymph node dissection (ILND) is performed because of the high risk of metastatic disease. In nonpalpable ILNs (cN0), the risk of micrometastatic disease is 20% [1], and a number of therapeutic options can be utilised to detect occult disease [2]. At present, currently available noninvasive staging techniques still lack sufficient accuracy to reliably stage the nodal status. Therefore, invasive surgical staging (modified ILND or Dynamic sentinel lymph node biopsy (DSLNB)) still remains the technique of choice in this challenging group of patients, who are at high risk of micrometastasis. However, performing ILND in men with cN0 disease even at high risk of micrometastasis will result in overtreatment of 80% of patients [3] and is associated with significant morbidity [2]. In order to improve detection rates, risk stratification based upon the histology of the primary tumour and less invasive methods have been introduced. The EAU guidelines advocate that, in men with cN0 low risk (Tis, TaG1, and T1G1) disease, only surveillance is recommended. In higher risk (≥T1G2) disease, invasive lymph node staging by either bilateral-modified ILND or DSLNB has been recommended as the diagnostic staging modalities of choice [2]. DSLNB is easy applicable, minimally invasive, with 88% sensitivity and 90% specificity as demonstrated in meta-analysis [4]. The main disadvantage is a false negative rate of up to 18%. It means regional inguinal recurrence after excision of a tumour-negative sentinel lymph node [2, 3, 5]. Other disadvantages of DSLNB include nonvisualisation, which can be resolved with immediate or deferred modified ILND (MILND) introduced by Catalona in 1988 [6]. A refinement of the histopathologic analysis of SNs (via serial sectioning) and mainly surgical exploration of scintigraphically nonvisualized inguinal basins decreased the overall false negative (FN) rate from 19.2% to 4.8% [1]. FN can be reduced to 5.3% by combination ICG-^99m^Tc-nanocolloid and with tracer reinjection up to 2.1% (16/756) [7]. Therefore, the main concern remains a risk of false negatives (and subsequent development metastatic disease) which can be potentially reduced even more by replacement of DSLNB by MILND [3, 6]. However, even modified ILND (i.e., less extensive) is associated with significant morbidity. Morbidity can be further reduced by a minimally invasive, endoscopic = laparoscopic approach. This technique is called VEILND (video endoscopic LND), which is performed by standard laparoscopic instruments or with a robotic system [8–14]. The visualisation and subsequent reliable removal of SLN can be performed by implementation of fluorescence imaging with indocyanine green (ICG) [15, 16]. The aim of this study was to validate the applicability of a combination ICG marking of SLN in VEILND and determine the optimal timing and dosage of ICG.

## 2. Materials and Methods

**Figure 1:**
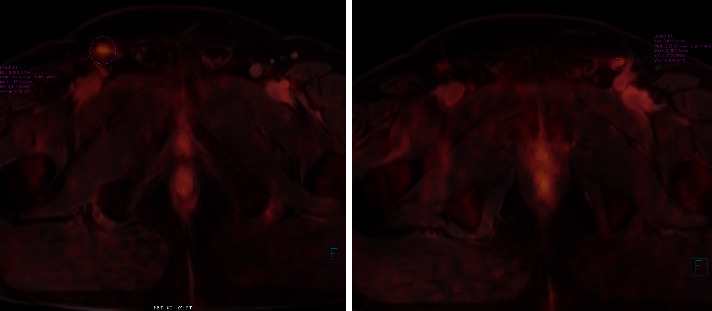
FDG PET MRI of both groins. A 69-year-old man underwent total penectomy for HPV-induced keratinizing squamous cell carcinoma pT2 G1 cN0 (nonpalpable LNs in groins), but positive on FDG PET MRI. VEILND with ICG on both sides performed, metastasis described on histopathology in SLN of the left groin. Maximal standardised uptake value (SUVmax) of penile cancer was 20.9; lymph node in the right groin 5.3, left 5.1. (a) To the distal part of penis. (b) To the prepubic area in a man with a history of total penectomy.

Between 3/2017 to 1/2021, 64 men were treated for penile cancer. Inguinal lymph node sampling was indicated in 26 of men with ≥pT1G2cN0. DSLNB was performed in 20 groins (2 with subsequent VEILND) and VEILND in 33 groins (7 unilaterally). VEILND (instead of DSLNB) was indicated in men with a higher risk of metastatic disease (≥T3, G3, nonpalpable ILNs but active on FDG PET MRI; PET MRI is routinely performed in all penile cancer patients at our centre [17]) (Figure 1). In 10 patients with VEILND (16 groins), ICG application with fluorescence near-infrared (NIR 803⟶830 nm) detection was undertaken. Surgery was performed in the supine position (“frog leg”). ICG was applied subcutaneously adjacent to the penile lesion or to the residual stump of the penis or to suprapubic region (in men with a history of total penectomy: 5 cases).

**Figure 2:**
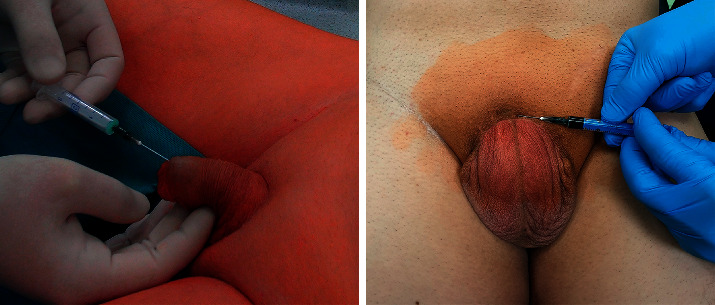
Subcutaneous application of 2.5 mg ICG (in 1 mL).

**Figure 3:**
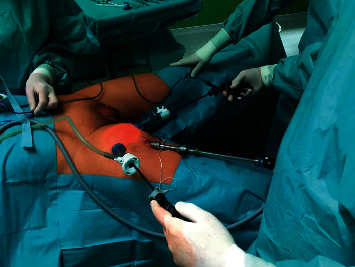
Right side VEILND (video-endoscopic lymph node dissection)—camera 0° and 2 ports 5 and 11 mm with fixation balloons (Kii^Ⓡ^ Advanced Fixation, Applied Medical).

**Figure 4:**
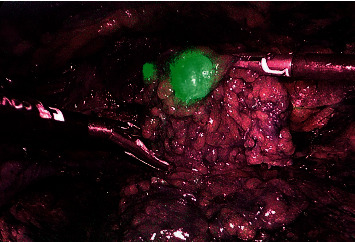
Green-coloured sentinel lymph node visible during laparoscopy visualised by ICG application and fluorescence near-infrared (NIR 803 ⟶ 830 nm) detection.

**Figure 5:**
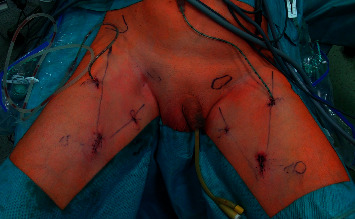
A man just after bilateral VEILND with ICG left. Femoral triangles, suspicious sites of sentinel lymph nodes and sites and sizes of three ports were marked with pen before surgery.

Surgical technique: ICG was applied immediately prior to surgery under general anaesthesia (Figure 2). The region of SLN was determined after 20–30 min, the contralateral side was performed at least one hour after application. The dose of 1.25 mg ICG (Verdye®) diluted in 2 mL was applied in one case with invisible SLN, the dose of 2.5 mg in diluted in 1 mL was applied in 8 cases, and the dose of 5 mg diluted in 2 ml was applied in the remaining 6 patients (10 groins). Surgery was performed within the boundaries of the femoral with a SLN located above inguinal groove close to the penis. After making a small incision on the superior margin of the femoral triangle, an index finger was placed to create a space under Camper's fascia. 2 ports, 5 and 11 mm (trocar with fixation balloons), were introduced through the incision videoport, (optic 0°, CO_2_ pressure at 12 mm Hg) (Figure 3). The great saphenous vein was preserved. Lipolymphatic tissue was harvested from the Daseler zone 1, 2, and 5 (around and in the fossa ovalis, inguinal ligament, only above the confluence of great saphenous vein with femoral vein, under Camper's fascia and caudally to inguinal ligament) with Ligasure® Maryland (or Blunt tip 5 mm). SLN was visualised with fluorescence near-infrared detection in order to increase the likelihood of removing the actual SLN, which may be left unexcised (Figure 4). Lipolymphatic tissue was extracted in an Endocatch® bag Gold or directly through the incision without a bag. A suction drain was placed, and port sites were closed (Figure 5). Compressive dressing of the groin was applied for 24 hours. A single dose of antibiotic prophylaxis (coamoxicillin) was given prophylactically and eventually after 8 hours. Venous thromboembolism prophylaxis was routinely given. Mechanical compression stockings of lower limbs, miniheparinization (low molecular heparin), was given for 3 weeks.

## 3. Results

VEILND was performed in 33 groins (20 men), with ICG in 15/24 (See Table 1 for details). The great saphenous vein was not preserved in 4 groins (12.1%). Metastatic disease (pN+) was detected in 9 of 33 groins (27.3%) at final histopathology. A single positive LN was detected in 7 groins, two positive LNs were noted in 1 groin and four positive LNs in the remaining groin. Complications occurred in 13 of 33 groins (39.4%). Failure of marking SLN with ICG was observed in 25.0% (6/24). One was due to the application of 1.25 mg ICG only (first case); one was due to extensive metastasis to SLN; in four cases, the cause was unknown (16.7%–4/24). In the short follow-up period, no local recurrence was seen in the pN0 ICG group. Progression of disease in the ICG group was seen in 5 men with pN+.

## 4. Discussion

The main goal of this study was to add marking of SLN with ICG to an already standardised VEILND, with the main aim of the study to determine optimum timing of application, dosage, and outcomes of SLN labelling.

The concept of sentinel LN (SLN) in penile cancer was described in 1977 by Cabanas [18]. Detailed anatomy of inguinal lymphatic drainage with a variety of mapping substances in a feline model was performed in 1991 with optimal results with isosulfan blue [19], which enabled the application of DSLNB in humans. For surgeons, reliable marking of the SLN is crucial. The first method of marking of the SLN was introduced in melanoma with the use of isosulfan blue in 1992 [20] and with radiocolloid (^99^mTc-nanocolloid) detected with gamma probe in 1993 [21]. Application of concept of DSLNB marked with radiocolloid and portable gamma probe in penile cancer was popularised by Horenblas et al. in 2000 [22]. Jakobsen et al. determined the diagnostic accuracy of DSLNB in 222 men (409 groins); eight were false negative with a sensitivity of 89.2% (95% confidence interval 79.8–95.2%) per groin. Interestingly, four of 67 (6.0%) T1G1 patients (generally recommended for active surveillance only) had a positive SLN. In all, 28 of 222 (13%) patients had complications of Clavien–Dindo grade I–IIIa. Inguinal LN dissection was avoided in 76% of patients [23]. Lam et al. published their experience of 500 cases of DSLNB with false negative rate in 5% of groin basins [24]. Our experience with DSLNB in 68 groins led to [5] nonvisualisation in 17.6%, with the result that nearly 20% of men undergoing DSLNB were converted to MILND. Metastatic disease developed in pN0 (sn) in 5.6% (5/89 groins) (unpublished data).

The concept of marking SLN with ICG was first described in breast cancer in 1999 [25], although the systematic application of ICG was performed in 2012 [15, 16]. To enhance detection of disease, both radiocolloid and ICG are combined in hybrid tracer ICG-^99^mTc-nanocolloid [7, 15, 26]. Marking with ICG can be combined with patent blue [27, 28] as well, and SLN can be visualised without fluorescence near-infrared (NIR) camera by standard laparoscopic optics. In centres not equipped with NIR technology, patent blue only can be an option for the SLN marking. At our centre, patent blue is used in open DSLNB only. Chemical characteristic of molecule of ICG can explain some mistakes in diagnostics. The small molecule of ICG (and patent blue as well) flows through nodes quickly, and that a fluorescent node is not necessarily the “true” SLN, as another fluorescent node may be obscured by overlying fat since the tissue penetration depth of the fluorescent signal is still limited (that is why, a radiocolloid, or combined ICG with radiocolloid, is still the standard). It can lead to risk removing unnecessary number of nodes or removing a higher tier node instead of the SLN or not finding the SN (exemplified by high nonvisualisation rate of 25%).

Before widespread implementation of VEILND with ICG, there remain some important technical questions: dosage, concentration, and timing of ICG application. In vulvar cancer, gynaecologists use 1 mL ICG (0.5 mg/mL) and for uterine and cervical malignancies 4 cc (1.25 mg/mL) injected into the cervix [28]. For marking the SLN in different urological malignancies, the recommended dosage is 2 cc, 2.5–5 mg/ml [29]. Contraindications to ICG application is allergic to iodides. Local injections of ICG into the tumour results in rapid lymphatic clearance (within 15 min), thereby mapping the lymphatics drainage patterns from the injection site [29]. Therefore, perioperative application is optimal.

As discussed, there remains a dilemma of how to reduce the false negative rate of DSLNB and how to solve nonvisualisation in DSLNB is MILND. The lateral and caudal extents of nodal excision are reduced, and the saphenous vein is preserved. Based on our experience, metastasis to the caudal part of the groin (Daseler zones 3 and 4) in a generally recommended template for radical ILND are extremely rare, and this region can be avoided even in radical ILND. The original template was designed with reference to melanoma which has a different distribution of metastases. The main goal of MILND is to reduce the risk of complications such as wound infection and lymphedema [6], which can occur in up to 55% of patients. Minimally invasive LND (laparoscopic/robot assisted) further reduces this morbidity [30, 31]. In our opinion and experience, ICG marking seems to be the optimal method for routine clinical practice. Near-infrared (NIR) fluorescence imaging using the NIR fluorescence agent ICG enables real-time intraoperative visualisation of the SLN.

Gynaecologists have compared VEILND with conventional open inguinal LND in women with vulvar cancer: the lymph node yielded 15 ± 5 vs 18 ± 6, *P*=0.058), with a similar 2-year recurrence rate (10.5% vs. 10%, *P*=0.957) and 2-year disease-specific survival rate (95.5% vs. 93%, *P*=0.724). In our group of modified VELIND (it means less extensive than radical), there was gain of lymph nodes significantly lower, mean 8.1 LNs only (Table 1). The wound complication rate was significantly lower in VEILND: 4.8% vs. 55.6%, *P* ≤ 0.001, whereas body image scores and cosmetic scores were higher [32]. Master et al. in a series of 41 VEILND performed in melanoma and penile cancer patients described complications as documented by Dindo–Clavien I in 2.6%, II in 24.4% and IIIa in 21%. Superficial wound infection occurred in 2.6%, lymphocele in 12%, mild-moderate lymphedema in 12%, readmission for i.v. antibiotics in 10.5% and flap necrosis in 2.6% of cases. No skin edge necrosis was detected. Median operative time range was 75–398 (175) minutes [30]. Yadav et al. found postoperative complications in 34.48% in the open ILND and 10.34% in VEILND, all performed for penile cancer only. The complications of 29 VEILND vs. 29 open ILND were skin necrosis (all grades) 2 vs. 8, lymphedema 3 vs. 4, lymph collection 3 vs. 3, and wound infection 0 vs. 4. The mean operative time was 162.83 vs. 92.35 minutes, and the mean numbers of removed lymph nodes were 7.6 vs. 8.3 [11]. Singh et al. compared robot-assisted VEILND vs. open ILND: operative time per limb - median 75 vs 60 minutes, *p* < 0.0001, incidence of major complications 2% vs. 17%, *p*=0.0067, edge necrosis 9.8% vs. 23%, *p*=0.048, flap necrosis 2% vs. 13%, *p*=0.035 and severe limb oedema 0% vs. 9%, *p*=0.029. The groups experienced a similar incidence of lymphocele, surgical site infection, cellulitis, and early and late limb oedema [31]. A high percentage of lymphorrea and especially later developed lymphoceles in our group is speculatively attributed to the necrosis of primarily closed lymphatic vessels (with sealing instrument) that have opened secondarily.

## 5. Conclusion

In this study, we have attempted to standardize VEILND with florescence indocyanine green (ICG) marking of SLN in penile cancer ≥ pT1G2 and cN0, including timing of application and dosage of ICG. Based on our experience, we can conclude the following: (1) laparoscopic MILND is feasible and reproducible (2) with still relatively high risk of morbidity (especially lymphocele in >40%). Because of price and high rate of complications of VEILND, DSLNB should be remained gold standard; (3) ICG can be safely applied and must be detected with special technology NIR fluorescence imaging; (4) nonvisualisation is found especially in extensive metastatic infiltration of SLN; (5) ICG can be applied just before surgery (6) in dose at least 2.5 mg diluted in 1 mL (better 5 mg in 1 mL) and (7) in man with previous total penile amputation to the prepubic region. Further studies are needed to validate this technique in the detection occult metastasis in men with cN0 penile cancer.

## Figures and Tables

**Table 1:** Results of VEILND (video endoscopic inguinal lymph node dissection) simple and with florescence indocyanine green (ICG) application.

	All VEILND	VEILND *w* ICG
No of men	20	15
No of groins^*∗*^	33	24
Failure of ICG marking		6 (25.0%)
Age†	63.4 ± 9.9 (41–78)	61.6 ± 10.6 (41–78)
Time of surgery per groin†	63.8 ± 15.3 (43–92)	59.5 ± 11.2 (43–81)
BMI†	32.7 ± 6.6 (24.5–50.9)	33.7 ± 6.6 (25.7–50.9)
Number of excised lymph nodes†	8.1 ± 3.7 [3–20]	8.5 ± 4.0 [3–20]
Number of groin with positive lymph nodes	9/33 (27.3%)	9/24 (37.5%)
Mean hospital stay†	16.8 ± 10.4 (6–44)	16.5 ± 9.0 (6–39)
Complications Dindo–Clavien I-II^*∗∗*^	12 (36.4%)	10 (41.7%)
Complications Dindo–Clavien I-II^*∗∗∗*^	1 (3.0%)	0 (0.0%)

*Notes*. ^*∗*^7 cases unilateral-contralateral side was done open 4x due to cN1, two were not marked as SLN (the other done DSLNB) and the last was pN1 (sn) on one side by the primary DSLNB), and this VEILND did without ICG. ^*∗∗*^lymphoceles, ^*∗∗∗*^revision for bleeding with subsequent skin necrosis. †mean, STDEV (standard deviation), minimal and maximal value.

## Data Availability

All data are available in the hospital system of University Hospital Plzeň, CZ.
